# The Experience of Dutch Women Using a Coping Intervention for Oocyte Retrieval: A Qualitative Study

**Published:** 2020

**Authors:** Henrietta D.L. Ockhuijsen, Ida Ophorst, Agnes Van Den Hoogen

**Affiliations:** 1- Department of Reproductive Medicine and Gynaecology, University Medical Centre Utrecht, Utrecht, the Netherlands; 2- Department of Children, Princes Maxima Centre and University Medical Centre Utrecht, Utrecht, the Netherlands; 3- Department of Neonatology, Wilhelmina Children’s Hospital and University Medical Centre Utrecht, Utrecht, the Netherlands

**Keywords:** Anxiety, Early intervention, Fertilization *in vitro*, Oocyte retrieval, Pain, Psychological adaptation

## Abstract

**Background:**

Many women experience oocyte retrieval during an IVF treatment as a stressful and emotionally difficult situation. Women fear the pain as associated with oocyte retrieval. Based on the existing literature, a coping intervention for oocyte retrieval (CIFOR) was developed to deal with the stress and pain during oocyte retrieval. The objective of this study was to explore the experiences of women using coping intervention for oocyte retrieval (CIFOR) while undergoing oocyte retrieval.

**Methods:**

For this generic qualitative study, a purposeful sample of fifteen women was gathered from a university clinic in the Netherlands and each participant was interviewed. Background information about the IVF treatment was collected from medical files. Semi-structured interviews were performed approximately 15 *min* after the OR procedure. Data were analyzed using the Qualitative Analysis Guide of Leuven and processed using MAXQDA.

**Results:**

Twenty-five women were approached for this study between January and May 2018. This study identified five themes that were important in the experiences of women using CIFOR: highly valuing the CIFOR, feasible in daily practice, need for information, sense of control and partner’s involvement.

**Conclusion:**

Women highly valued the tool. They found CIFOR feasible in daily practice and it fulfilled their needs for information. In addition, women had a sense of control using the intervention. Future research will involve performing a pilot study according to the Medical Research Council framework with outcomes based on the patient’s sense of control, ability to cope, coping strategies, anxiety and pain.

## Introduction

In Europe, it is estimated that one or two women out of 100 between the ages of 20 and 44 cannot become pregnant with their first child (1). Ten out of 100 women who have already a child, have problems to become pregnant for a second time (1). The most common causes of infertility are ovulation disorders (24%), a reduced quality of semen (20%), disorders in interaction of semen and cervix mucus (15%) and tuba pathology such as endometriosis (11%). In 30% of all cases, the cause is unknown (2). One treatment for infertility is *in vitro* fertilization (IVF), also called test tube fertilization. IVF is a reproduction technique whereby one or more oocytes are fertilized with sperm outside the body. The resulting embryo or embryos are then placed into the uterus. According to estimates of the European Society of Human Reproduction and Embryology (ESHRE), 350,000 babies per year are born worldwide through IVF (3).

The most painful part of IVF treatment is the oocyte retrieval (OR). A cohort study of women receiving an IVF treatment showed that 6.9% of 743 women found it to be very or extremely painful (4). Several psychological factors such as anxiety, side effects of hormonal treatment, previous negative experiences with gynaecological examinations and perceived lack of control may be related to the pain experienced during OR (4). Anxiety has been described as being associated with a lower pain threshold and the feeling of being in control has been associated with the ability to cope with pain more efficiently (5).

Several studies have been performed relating to different methods of pain relief during OR, such as conscious sedation, analgesia, electro acupuncture and paracervical block (6, 7). They concluded that no method was superior to the others, and no consensus was reached on optimal pain relief during OR. It was advised that pain relief should be determined on an individual basis because non-physical factors, such as motivation, the ability to cope and the medical team’s support, likely influence the experience of pain (6).

Research on coping and psychological interventions before OR is limited. However, a recent RCT found that music therapy during oocyte retrieval significantly decreased the vaginal pain (8). Based on an unpublished mixed method study and the existing literature about coping, a coping intervention for oocyte retrieval (CIFOR) was developed by a psychologist (9). This mixed method study interviewed 31 women (1st IVF cycle=11, 2nd=13, 3rd=3, >3rd=4) post OR. The anxiety level was higher for women who experienced their first IVF cycle (9). The women were given information about OR, and they considered IVF a helpful approach. The women were anxious for several reasons, including worries about the number of eggs available (40%), level of pain (23%), the unknown (10%) and fear of needles (3%). Based on this study, Newton concluded that giving women pre-IVF information about possible outcomes like amount of eggs, level of pain, and quality versus quantity of the oocytes may be helpful (9). Women reported that they used several self-generated coping techniques, *i.e*., relaxation (Such as deep breathing, imagery and positive self-talk) (56%), distraction such as watching the monitor (37%) and distraction/affiliation such as focusing on husband’s face or hand (23%). External facilitators included nurse behavior (Small talk, reassurance), physician behavior (Encouragement, explanation), lighting (67%), music (47%) and feedback about eggs retrieved (25%).

CIFOR is based on the framework of stress and coping developed by Lazarus and Folkman (10). They define coping as a constant shift in cognitive and behavioral efforts to manage specific burden-some situations. Patients have limited control during OR because of restrictions on movement, the unknown length of the procedure and uncertain procedural outcomes. It was expected that if patients were given control over minor but seemingly important aspects of the OR, they would cope better with the procedure (10).

In the Netherlands, CIFOR has never been used; therefore, it is important to gain an understanding of the experience of Dutch women who have undergone an OR using this coping intervention. This is a complex process, and the elements detailed by Medical Research Council, such as the development and feasibility of the coping intervention must be carefully taken into consideration (11). This coping intervention requires further modeling and development before it can be implemented in the future and before determining whether such intervention is feasible and useable for Dutch women who undergo oocyte retrieval. The aim of this study was to explore the experiences of women using CIFOR while undergoing oocyte retrieval. A qualitative study was conducted to investigate these experiences.

## Methods

### Design:

A generic qualitative study was performed (12, 13) to focus on how people interpret their experiences and what meaning they attribute to their experiences (14, 15). This study was conducted between February and June 2018.

### Sample:

To gain insight into different perspectives while choosing informative cases (16), a purposeful sample of women with maximum variation was selected by the researchers from the medical files of a fertility clinic at the University Medical Centre Utrecht. Inclusion criteria were women who were undergoing their first, second, third or fourth IVF treatment. The women differed in age, and had varying quantities of follicles and children. Women were excluded if they did not speak Dutch, or if they underwent an OR for social and medical freezing or donation, because participation in this study could be viewed as a burden by these women.

### Oocyte retrieval:

In total, the oocyte retrieval procedure lasted 30 *min* and the suction to get the oocytes out of the ovaries lasted 5–10 *min*. The women undergoing the oocyte retrieval received paracetamol and diclofenac suppository one hour before the procedure. A transvaginal ultrasound guided oocyte retrieval was used to collect the oocytes from the ovary. The vaginal wall was numbed by a local anesthesia. A physician inserted a needle through the vaginal wall into an ovarian follicle. The needle was attached to a suction device.

### Intervention:

The CIFOR consists of a booklet in three parts. The first part gives pictures and detailed information about the procedure, pain related information (Description of physical sensetion and pain intensity ratings) and outcome information (Average number of eggs retrieved, quality versus quantity of oocytes). The second part gives information about different coping strategies to be used during the OR (Muscle relaxation, deep breathing, distraction techniques and positive reappraisal). The third part is a personal coping plan which consists of four main categories of sense of control, distractions, self-talk and environment.

Women received verbal and written information about the intervention at the beginning of the treatment from a nurse. During the treatment, it was always possible to contact a nurse in case of questions about the CIFOR. Women had to complete a personal coping plan before the egg retrieval. On the day of the OR, the plan was handed over and discussed with the physician and the nurses who were present during the retrieval.

### Data collection:

An independent member of the treatment team contacted the selected women by telephone. Women who wanted to participate received verbal and written information concerning the coping intervention from the researcher. In the subsequent days leading up to the OR, the women could read the information and fill out the personal coping plan. On the day of the OR, the plan was handed to the attending physician and nurse, who ensured that the coping plan was executed correctly during the procedure. Data collection continued until data saturation was achieved. Fifteen interviews were conducted, and saturation was reached after 12 interviews. Three additional interviews were performed to confirm saturation. This meant that data saturation was reached when there was sufficient information to replicate the study, no new information was obtained and coding was no longer feasible (17). Interviews were conducted by a female student researcher. The student researcher followed a training course on interview techniques and conducted two test interviews. Semi-structured interviews were performed 15 *min* after the OR procedure, before the women received information about the number of oocytes collected and semen quality. Therefore, the interviews were not influenced by the patients’ positive or negative reactions to this information. Interviews were digitally recorded, and a full transcription was made. A pre-prepared interview guide was created, consisting of three main topics: how did you experience the oocyte retrieval?, how did you experience CIFOR and how did you experience the support of the physician or nurse?. The researcher used a visual analogue scale (VAS) to determine pain and anxiety rates (18). Information about characteristics including age, diagnosis, number of children, quantity of follicles and education was collected from medical files and from semi-structured interviews.

### Data analysis:

**Figure 1.**
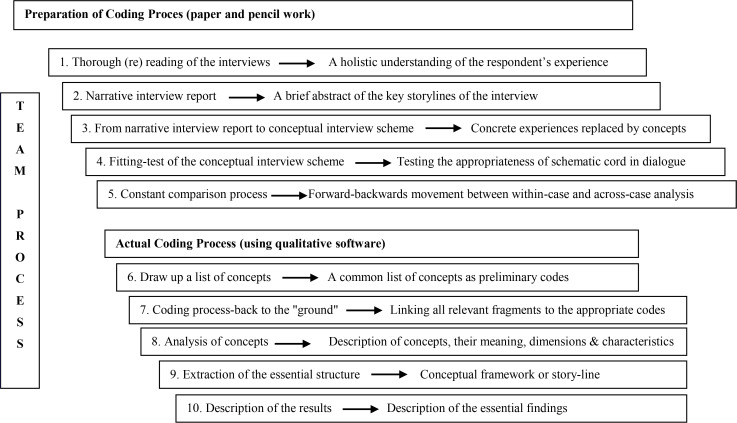
Qualitative analysis guide of leuven

The interviews were transcribed verbatim and analyzed using the Qualitative Analysis Guide of Leuven (QUAGOL) (19). This guide consists of 10 stages covering the preparation of the coding process and the actual coding process (Figure 1), for which a qualitative software programme (MAXQDA 10) was used. The transcriptions were read and coded by IO and HO. Data collection and analysis is an iterative process, in which there is constant forward and backward movement between collection and analysis. As interviews progressed, more themes were identified. Our focus was on gaining in-depth information from women regarding their experience of the OR while using CIFOR. To attain further in-sight into this data, when there was no agreement between IO and HO, essential and common themes were discussed by the research group. To guarantee validity and trustworthiness, two researchers were involved in the process of coding and the discussion of themes and conclusions. Furthermore, short reports and observational memos were written regarding the interviewees and the context of each interview so that the researcher could understand the interviews in their particular contexts. Credibility was taken into account by making the findings compatible with participant perceptions (20).

### Ethical consideration:

The Medical Research Ethical Committee of the University Medical Centre of Utrecht approved the research proposal. A consent form was signed by all research participants. Confidentiality of data and records was maintained by using numbers and fictional names.

## Results

### Recruitment and socio-demographic characteristics:

Twenty-five women were approached for this study between January and May 2018, and 15 wanted to participate, gave their consent and were interviewed. The interviews lasted between 26 and 39 *min*. All partners were present during the interviews. Women chose not to participate due to the following reasons: they did not speak Dutch, they were experiencing emotional or relationship problems, they were not motivated to participate in research, the intervention had no added value or did not fit with their own coping strategies or the treatment was postponed due to a lack of follicles.

The socio-demographic characteristics of the women who participated showed that maximum variation was achieved in age, number of treatments, diagnosis, number of children and educational level (Table 1). This diversity is relevant to understanding the experiences of different women.

**Table 1.** Socio demographic characteristics of participating women

**Name**	**Age (years)**	**Children (n)**	**Diagnosis**	**IVF treatments (n)**	**Follicles (n)**	**Anaesthesia**	**Education**
**Marieke**	35	1	PGD	1	11	SUP, LA	Unknown
**Christa**	40	1	Tuba anomaly	2	6	SUP, LA, SA	University
**Lynn**	36	0	Azoospermia	3	11	SUP, LA, SA	University
**Wendy**	27	0	Endometriosis	1	13	SUP, LA, SA	Lower secondary general
**Benthe**	27	0	Azoospermia	1	9	SUP, LA	Higher vocational education
**Julia**	33	0	Azoospermia	1	12	SUP, LA, SA	University
**Meral**	20	0	Azoospermia	3	13	SUP, LA, SA	Higher vocational education
**Haife**	30	0	PGD	3	17	SUP, LA, SA	Intermediate vocationaleducation
**Valerie**	32	0	PGD	2	11	SUP, LA, SA	Higher vocational education
**Xandra**	28	0	PGD	2	11	SUP, LA	Higher vocational education
**Anne**	38	1	Unknown fertility problem	2	13	SUP, LA, SA	Higher vocational education
**Patricia**	34	1	PGD	3	6	SUP, LA, SA	Higher secondary general
**Janet**	41	0	PGD	3	3	SUP, LA, SA	University
**Sofie**	34	1	Male factor	3	15	SUP, LA, SA	Intermediate vocational education
**Suzan**	41	1	PGD	4	9	SUP, LA, SA	University

PGD=Pre Implanted Genetic Diagnostic, SUP=Suppository, LA=Local Anaesthesia, SA=Systemic Anaesthesia

### Themes:

Five themes emerged from the interviews: highly valuing the CIFOR, feasible in daily practice, need for information, sense of control and partner’s involvement. Each of these themes will be described and substantiated by quotes from the participants.

### Highly valuing the CIFOR:

This study found that all women were positive about using CIFOR. Most of the women who were undergoing an OR for the second, third or fourth time already had found a way to cope with the procedure and suggested that CIFOR was more valuable when preparing for the first OR. The women’s experiences of pain and stress varied.

Overall, 12 of the 15 women who underwent an OR got through the procedure well. Women who already had received an OR said that they experienced less pain and were less anxious than in earlier treatments. Three women who were undergoing their first OR had underestimated how painful it would be.

“I have to say, I did not prepare myself very well … . I thought those breathing exercises were unnecessary for me, because so far the examinations for the IVF treatment were quite easy to handle, but the OR was pretty painful” (Benthe, 27, 1st IVF).

Despite pain medication, various pain scores were reported during the interview. On the pain VAS ranging from zero to ten, scores for local anaesthesia of the vagina were between one and seven, ovary puncture between one and nine and suction of follicles between zero and seven. Most of the women found the follicle suction more painful than the local anaesthesia. Local medication, as well as a systemic anaesthetic injection with morphine, was given to 12 women.

“I especially liked CIFOR because actually it is the most important part in your life ... That is how we experience it … and you actually give up on the fact that you cannot get pregnant without help of the hospital and doctors or whatever you need and that is a big downer. By using this you can make it just a bit more personal” (Wendy, 27, 1st IVF).

### Feasible in daily practice:

All women were positive about CIFOR’s feasibility. A majority were able to use CIFOR while preparing for and during the OR. Nurses discussed the coping plan with the women before the OR.

“To fill in the coping plan was not that difficult or special. I became more aware of the possible coping strategies. I liked that. So I thought what can I do and what do I like and how can I get through this procedure …” (Suzan, 41, 4th IVF).

Three women suggested that it might be better to link the information from the brochure to the coping plan more clearly.

“I have to fill out the coping plan, and it should match more with the information brochure. Here (Indicates the coping plan) it says breathing and relaxation exercise and in the brochure, it is written another way. Sometimes I wanted to know more about a strategy, and then I had to search for it in the brochure. I think it should be easier when you have got titles in the coping plan and brochure which are comparable with each other. Then it makes it easier to look back and fill everything out” (Meral, 20, 3rd IVF).

The interval between when the women were given detailed verbal information and their OR process ranged from two to nine days. This was enough time for the women to read and fill out the coping plan. The coping plan was filled out by 13 women at home. Twelve of these women brought it with them to the hospital and discussed it with the nurse present for the puncture.

“Yes, we went through it before start of the OR. Then we also discussed this and what I will arrange and that it will be all right. They [The nurse and doctor] both told me what they were doing and why, and it was what I expected” (Benthe, 27, 1st IVF).

One woman forgot to bring her coping plan, but told the nurse how she wanted to proceed. Another participant filled out the plan after the puncture, but had discussed her preferences with the nurse. One woman and her partner reported that filling out this coping plan had caused them stress due to the number of choices.

“You’ve got a lot of choices and suddenly you’ve got to think about that. Some things we already do, but maybe you just think about it a little bit more and that’s good too…” (Patricia, 34, 3rd IVF).

Others, especially women who were having their first OR, reported that filling out the coping plan before the procedure was difficult because it is hard to determine which coping strategies one wants to use and which are feasible.

“I found it difficult to fill out the plan because it is … well, I can say right now I do like to use that strategy and I suppose I will, but I might respond differently and say “just leave me alone” (Wendy, 27, 1st IVF).

### Need for information:

This study found that the majority of the women were positive about the informative brochure and coping plan. They found the brochure to be comprehensive and clear. Detailed information about the procedure was very helpful to read before the first OR.

“The general hospital brochure describes what you can expect, but this one was more detailed. It gave more information as to exactly what to expect. You are more prepared when the OR starts” (Sofie, 34, 3rd IVF).

Three out of four women who underwent their first IVF treatment found that using CIFOR was helpful in preparation. Some women read the brochure once or twice, and one read it more frequently, up to 20 times.

“I like the information brochure just because it gives detailed information about what you can expect during the puncture. It says what the physician and nurse are going to do, and I can say what I prefer” (Sofie, 34, 3rd IVF).

One woman had not realized what the puncture involved, and even with the provided information, she was not prepared and found it to be more painful than expected. A few women were upset by the detailed information and thought the images were too alarming which increased their anxiety.

“The information brochure was too threatening for me and too detailed. I knew about it, but I did not want to see it. I wanted a bit more superficial information. The pictures gave me a negative experience. I did not like them” (Haife, 30, 3rd IVF).

Most of the women said that the brochure reduced their stress because they knew what to expect.

“Well, I think it is pleasant, because women can prepare themselves better. They can be mentally prepared and go through the steps of the puncture in their mind, and so they can be more relaxed during the puncture and start it with more control. They know what to expect” (Janet, 41, 3rd IVF).

### Sense of control:

The use of CIFOR gave women a sense of control, not only over the OR, but also over which coping strategies suited them and should be used. There was some variation in the chosen coping strategies (Table 2).

**Table 2.** Results of the completed coping plan by the participating women

	**Marieke**	**Christa**	**Lynn**	**Wendy**	**Benthe**	**Julia**	**Meral**	**Haife**	**Valerie**	**Xandra**	**Anne**	**Patricia**	**Janet**	**Sofie**	**Suzan**	**Totaal**	**Totaal %**
**Sense of Controle**																	
I say when I am ready to start		1	1				1	1			1	1	1			**7**	**47**
I ask for breaks during the puncture		1					1					1	1			**4**	**27**
I want to be told when to expect discomfort		1	1	1	1	1	1	1	1	1	1	1	1	1		**13**	**87**
I want to watch the monitor: the nurse explains what’s there		1	1	1	1	1		1	1	1	1	1		1	1	**12**	**80**
**Distraction**																	
Activities are practised at home	1	1									1	1	1			**5**	**33**
Breathing exercise	1	1	1	1		1	1	1			1	1	1	1		**11**	**73**
Relaxation exercise								1			1	1	1			**4**	**27**
White knuckling				1		1	1					1	1	1		**6**	**40**
Focus on picture or object in the room							1			1			1	1		**4**	**27**
Talk about something positive							1	1	1	1		1	1	1	1	**8**	**53**
Visualize a relaxing place			1										1			**2**	**13**
**Self-Talk**																	
Positive reminders about			1				1	1	1	1		1				**6**	**40**
Procedure		1						1	1	1		1				**5**	**33**
Outcome		1	1	1				1	1	1		1				**7**	**47**
**Enviroment**																	
Temperature (Blanket)		1	1	1	1					1		1	1			**7**	**47**
Lighting			1										1	1		**3**	**20**
Music				1		1				1			1			**4**	**27**
**Total**	**2**	**9**	**9**	**7**	**3**	**5**	**8**	**9**	**6**	**9**	**6**	**13**	**13**	**7**	**2**		

The most used coping strategies were: “I want to be told when to expect discomfort (87%), watch the monitor (80%), breathing exercise (73%)”. The majority of the women reported that they chose coping strategies similar to what they used in daily life. Women did not choose new strategies.

“Yes, it is now a more reflective process, because otherwise it is only a physical experience. But now with all information it can be a conscious experience. I read the steps on paper and felt a kind of sense of control about the process. I suggest that especially women undergoing their first OR should read this information brochure, because the first was the most nerve-racking for me. I didn’t know how the procedure would go” (Janet, 41, 3rd IVF).

The women were aware of what they themselves could do when preparing for an OR. All the described coping strategies gave women the realization that they had a choice of whether or not to use them, and this choice provided the women with a sense of control over the situation.

“I have to say, using this coping plan calmed me. It was a very nice experience. It made the procedure more personal, and it was positive to have control in knowing, I could say what I preferred” (Wendy, 27, 1st IVF).

In total, 12 women indicated that they wanted to know when there might be pain during the puncture. They also wanted to use the distraction strategy “looking at the monitor” to see what the physician was doing, including the suction of the follicles.

“I really liked seeing when the needle went inside and seeing what was happening. I watched not only the monitor but everything around me, what the nurses were doing. At the moment the puncture became uncomfortable, I focused on the monitor and not on my pelvic floor” (Xandra, 28, 2nd IVF).

In this study, it was found that seven of the women discussed whether they or the doctor should decide when to start the procedure. They found it difficult to choose when to start and thought the doctor should be in charge.

“Since I know that I am going to postpone it, there must be someone who asks “Are you ready?” “Yes or no?”. Then, the choice is still mine’ (Wendy, 27, 1st IVF).

### Partner’s involvement:

All women participating in the study had a partner. Partners took a passive role in using CIFOR; it was important for them to “be there” to support their wives during the procedure. The brochure went unread by 13 partners either due to a lack of time or a lack of interest.

“No, I didn’t read the information brochure. I thought it was more for her” (Xandra’s partner, 28, 2nd IVF).

“I joked a bit, provided a bit of distraction and I said a couple of times “You are doing well” and that kind of thing. The best thing you can do is be there for her!” (Julia’s partner, 33, 1st IVF).

However, all the partners had discussed the coping plan and strategies with their wives to varying degrees and were prepared to do what suited the women.

“I am here to give her support and give her positive energy and compliments” (Meral’s partner, 20, 3rd IVF).

## Discussion

This study focused on women’s experiences during CIFOR. Five themes were identified: highly valuing the CIFOR, feasible in daily practice, need for information, sense of control and partner’s involvement.

Women highly valuated the CIFOR. In general, the experienced pain during ORs was less than anticipated or experienced in previous ORs.

This study showed that women find CIFOR feasible. The feasibility of an intervention is based on whether it is appropriate for further testing; it enables investigators to assess whether an intervention is relevant and workable (21). According to the results, in general, CIFOR is practical and easy to use and overall the participants experienced a positive effect. Women were interested in it and intended to use CIFOR when preparing for OR experiences. It was not considered a burden to complete the coping plan and use the coping strategies. Furthermore, CIFOR is in line with the procedures of nurses and physicians, who seek to prepare and support women. A point of interest is that women needed time to read the brochure, and they suggested linking the information from the brochure to the coping plan in a better way.

This study also showed that different patients require different amounts of information. Most women wanted information, but some found the information too detailed and became stressed. This could be explained by the fact that both “monitors” and “blunters” are part of the study population. Two psychological coping styles can be identified for dealing with threats, monitoring and blunting (22). Monitors are concerned about risks with regard to a health threat and attend to threatening information, while blunters avoid threatening information. A randomized control study about the effects of an information brochure on patients’ experience of gastrointestinal endoscopy for the first time showed reduced anxiety by those who were high monitors (23).

Another theme that has emerged involves control. This study revealed that women have different preferences when it comes to control. Coping with a situation involves attempting to control it by modifying the environment, changing the situation or managing behavior and emotions (9). CIFOR is based on the stress and coping strategy of Lazarus and Folkman (24). These experiences of control can be divided into objective and subjective types. Objective control refers to the actual controllability of outcomes. Subjective control refers to the perceived control or the estimation of available control (24). Women have limited control during the OR. By using CIFOR, women were more aware of their opportunities and possible choices for different coping strategies, which allowed them to gain a sense of control during the process. In most cases, the women chose coping strategies that were familiar to their daily lives. In the future, professionals could encourage and teach women to use new coping strategies as well.

Partner involvement is the last important theme. The partners were largely concerned with “being there”. Most did not play an active role in using CIFOR. They thought it would be more applicable to the women undergoing the procedure. Some of the partners were not present during the introduction of the study. This could have influenced their involvement. Men experienced the IVF procedure in a different way. Qualitative studies showed that many men characterize their own approach to IVF as scientific; although the men in this study indicated a strong interest in technology, they were only passively or not actively involved in the process (25–27). The researchers in these studies confirmed that partners were largely interested in being present, doing what the women wanted and providing emotional support. Similarly, in this research project, most partners did not feel the need to read the provided information, but they did want to be there for their partners and support them in their preferred ways. To increase partner involvement, more information about CIFOR should be supplied. Partners’ passive roles in CIFOR did not shed light on how each pair experienced the process of IVF treatment together. Studies have shown that infertile couples endure the difficulties of infertility by sharing their thoughts and feelings and supporting each other (28).

The strength of this study is in its use of maximum variation. Due to the diversity in the participating women, CIFOR is shown to be widely applicable, and this study has provided different perspectives on its usefulness. Interviews allowed for a deeper understanding of participants’ experiences. Holding an interview directly after the OR was an advantage because the quantity of the oocytes and quality of sperm were unknown; therefore, the interview was not influenced by emotions related to the success of the procedure.

Some limitations need to be taken into account. One limitation is that although women were willing to participate, IVF treatment is an emotional procedure, and therefore in some cases participating in the study was viewed as an added burden. Some women experienced pain during the interviews. Moreover, some felt anxious about the results of the retrieved oocytes and the quality of the sperm. This could have influenced the interview, because the couples were distracted and tense. CIFOR was not always optimally under- stood by partners. To maintain participation of partners, more attention should be paid to explaining to them how to use CIFOR.

This study has showed that women had positive experiences using CIFOR and felt it was valuable during a first OR but less useful during a second, third or fourth retrieval. This type of coping intervention is feasible in daily practice, but practical adjustments must be made based on the experiences of the women.

## Conclusion

In conclusion, CIFOR can be used by women who undergo an OR. Women highly valued the tool.

They found it feasible in daily practice and it fulfilled their needs for information. In addition, women had a sense of control using the intervention. It is unclear if CIFOR reduces pain and anxiety; therefore, future research into the effect of CIFOR on pain and anxiety is recommended. Future research will involve performing a pilot study according to the Medical Research Council framework with outcomes based on the patient’s sense of control, ability to cope and coping strategies.
